# Association of Material Deprivation Status, Access to Health Care Services, and Lifestyle With Screening and Prevention of Disease, Montreal, Canada, 2012

**DOI:** 10.5888/pcd13.160157

**Published:** 2016-09-29

**Authors:** Garbis A. Meshefedjian, Marie-Jo Ouimet, Louis-Robert Frigault, Viviane Leaune, Sadoune Ait Kaci Azzou, Marie-Ève Simoneau

**Affiliations:** Author Affiliations: Marie-Jo Ouimet, Louis-Robert Frigault, Viviane Leaune, Sadoune Ait Kaci Azzou, Marie-Ève Simoneau, Direction régionale de santé publique, Centre Intégré Universitaire de Santé et de Services Sociaux du Centre-Sud-de-l’Île-de-Montréal.

## Abstract

**Introduction:**

The objective of this study was to provide information on the effect of disparities in material deprivation, access to health care services, and lifestyle on the likelihood of undergoing screening for disease prevention.

**Methods:**

We used data from a probability sample (N = 10,726) of the Montreal population aged 15 years or older and assessed 6 dependent variables (screening for breast cancer, cervical cancer, colon cancer, blood glucose, and high blood pressure and receipt of the seasonal influenza vaccination), and 3 independent variables (disparities in material deprivation, access to health care services, and personal lifestyle habits). We used logistic regression to analyze data and determine associations.

**Results:**

Use of preventive health services increased as material deprivation declined, access to health care improved, and lifestyle habits became healthier. The combined effect of household income, an individual measure, and the material deprivation index (consisting of quintiles representing a range from the most privileged [quintile 1: best education, employment, and income] to the most deprived [quintile 5: least education, employment, and income]) an ecological measure, showed that having a Papanicolaou test was significantly associated with high annual household income (≥$40,000) even if the woman resided in a deprived neighborhood (quintiles 4 and 5 of the material deprivation index) (odds ratio [OR], 1.38; 95% confidence interval [CI], 1.04–1.84), whereas odds of having a mammogram or influenza vaccination were significantly associated with living in a privileged neighborhood (quintiles 1, 2, and 3 of the material deprivation index) even among people with a low annual household income (<$40,000) (mammogram: OR, 1.54; 95% CI, 1.00–2.38; influenza vaccination: OR, 1.31; 95% CI, 1.04–1.66).

**Conclusion:**

In addition to influencing lifestyle habits and access to health care services, disparities in material deprivation influence whether a person uses preventive health services. Public health professionals need to establish screening outreach programs in socioeconomically disadvantaged neighborhoods to enhance public participation in disease prevention programs and reduce disparities in health.

## Introduction

Chronic diseases are the leading causes of disease and death worldwide. The World Health Organization estimates that by 2020, 4 chronic diseases — cancer, cardiovascular disease, chronic obstructive pulmonary disease, and type 2 diabetes — will contribute to 73% of all deaths (1). In 2011, these 4 diseases accounted for about two-thirds (65%) of all deaths in Canada (2). Furthermore, in 2010, the total economic burden of illness was $192.8 billion in Canada, of which at least 50% was attributable to chronic diseases (3). Therefore, chronic diseases pose a significant demographic and economic burden on the population health.

The health care system strives to improve population health through the prevention and management of chronic diseases. Adherence to screening guidelines may prevent or delay the occurrence of some chronic diseases (4–6). However, many determinants influence screening participation. Among screening tests considered in this study (Box), participation rates for breast and cervical cancer screening improved if women had a usual source of health care, had health insurance coverage, felt susceptible to the disease, had a supportive social milieu, had access to community outreach programs, and if screening was recommended by physicians (7–9). Conversely, lack of knowledge about cancer and screening programs, fear and embarrassment, mistrust of the health care system, low socioeconomic status, old age, limited contact with medical service providers, and belief in predestination negatively influenced rates of screening participation (8,10,11). Similar variables influenced colorectal cancer screening rates; for example, compliance with fecal occult blood test (FOBT) screening increased among people as patient education, household income, knowledge about cancer, engagement in health promotion behaviors, contact with the family physician, and a physician’s recommendation to have screening tests increased (7,12,13). Likewise, rates of diabetes screening increased with age and obesity and among people with a family history of diabetes (14). Finally, significant associations were found between blood pressure screening and diabetes and between current tobacco use and alcohol consumption (15).

**Box. Preventive Health Care Measures and Recommended Frequency (4–6)**

**Table:** 

Measure	Target Population	Recommended Frequency
Mammography	Women aged 50 to 69 years with no cancer	Every 2 years
Papanicolaou test	Women aged 20 to 64 years with no hysterectomy and no cancer	Every 3 years
Fecal occult blood test	Population aged 50 to 74 years with no cancer	Every 2 years
Blood glucose test	Population aged 40 years or older with no diabetes and no heart disease	Every 3 years
Blood pressure measurement	Population aged 18 years or older with no hypertension and no heart disease	Every 12 months
Seasonal influenza vaccination	Population aged 60 years or older or younger than 60 years with chronic diseases	Every 12 months

In addition to screening tests for cancer, diabetes, and high blood pressure, seasonal influenza vaccination is an effective preventive health measure (16). Research showed that getting an influenza vaccination depended on having a positive attitude toward the protective effect of the vaccine and having had the vaccination in the past (17,18). Conversely, experiences with side effects of vaccination and access to the vaccination discouraged compliance (18).

Factors related to socioeconomic status, health care needs, accessibility of health care services, lifestyle habits, and the knowledge, beliefs, and attitudes of people have a significant effect on participation in screening. To our knowledge, no previous study has analyzed the simultaneous and independent effect of disparities in material deprivation, access to health care services, and personal lifestyle habits on screening for breast, cervical, and colorectal cancer and diabetes and on receipt of seasonal influenza vaccination. The objective of our study was to examine the effect of 3 explanatory variables — disparities in material deprivation, access to health care, and personal lifestyle habits — on the likelihood of participation in cancer screening and influenza prevention to provide information to guide public health interventions at the local level.

## Methods

We examined a sample of 10,726 people (4,807 men and 5,919 women) taken from the estimated 1,650,000 inhabitants of Montreal, Canada, in 2012. We extracted data for this study from the 2012 edition of Montreal’s Local Health Survey Program (TOPO). We collected information on chronic diseases and their major determinants and risk factors: social conditions (employment, education, immigration, material deprivation), lifestyle (smoking, physical activity, food consumption), and the use of health care services. The study was approved by the Quebec Provincial Public Health Ethics Committee. Data were collected from February 27, 2012, through November 11, 2012, under the supervision of Le Secteur Surveillance de l’État de Santé à Montréal (Health Surveillance in Montreal, Canada).

Our sample was extracted in 3 waves from the Quebec Health Insurance Registry (RAMQ). RAMQ covers more than 95% of the Quebec population aged 15 years or older and contains data on sex, date of birth, home address, daytime and nighttime telephone numbers, and names of people living at the same address. First, we stratified the sample by 12 local health units in Montreal and by sex and 7 age groups (15–24 y, 25–34 y, 35–44 y, 45–54 y, 55–64 y, 65–74 y, ≥75 y). Then, we drew a stratified random sample (n = 28,940) from the RAMQ database by setting the number of respondents to 900 per local health unit on the basis of a minimum event prevalence of 5%, a maximum coefficient of variation of 15%, a response rate of 45%, a design effect of 1.1, and an eligibility rate of 90%.

After sample extraction, personalized letters were sent to the postal address of all people in the sample explaining the purpose of the survey and inviting them to access the questionnaire on the Internet. Subjects were followed up with telephone calls during the data collection period to confirm receipt of the invitation letter and to complete the survey over the telephone if the recipient did not intend to complete it online. Detailed information on the survey process is available elsewhere (19).

Data were adjusted for nonresponse. We used demographic information on respondents and nonrespondents in the sample to form homogeneous groups by using the χ^2^ automatic interaction detector method where the adjustment was done for each created group. The final sample (n = 10,726) was weighted to make statistical inferences to the estimated 1,650,000 inhabitants of the city. The response rate (41.4%) was computed by using the standard definition of the American Association for Public Opinion Research (20,21).

### Variables

This study considered 6 dependent variables and 10 independent variables. Dependent variables were being screened for breast cancer, cervical cancer, colon cancer, diabetes, and high blood pressure and receipt of seasonal influenza vaccination. Adherence regimens for these screening tests and vaccination (Box) were based on the recommendations of the Canadian Task Force on Preventive Health Care, the US Preventive Services Task Force, and the Centers for Disease Control and Prevention (4–6). The 10 independent variables were grouped into 3 explanatory variables and 7 controlling variables. The 3 explanatory variables were 1) disparities in material deprivation (combining household income and material deprivation index), 2) access to health care services (combining access to usual source of health care and having a family physician), and 3) personal lifestyle habits (combining smoking, physical activity, and daily fruit and vegetable intake). The 7 controlling variables were 1) sex, 2) age, 3) education, 4) language spoken at home, 5) immigration status, 6) obesity, and 7) presence of physical and mental illnesses. 

The material deprivation index, defined as a measure of socioeconomic conditions at the neighborhood level, was adopted from the literature (22). Its computation was based on the education, employment, and income characteristics of the smallest geographic area for which all census data were available. These areas were composed of one or more neighboring census blocks with a population of 400 to 700 persons within relatively stable census tracts as defined by Statistics Canada (23). Hence, the material deprivation index, was “not an individual measure of socioeconomic conditions, but rather a measure of the conditions seen at the neighborhood level” (22). The index was carried out using principal component analysis in each geographic area and the factor score of the components were ranked into quintiles ranging from the most privileged neighborhood in terms of education, employment and income (quintile 1) to the most deprived (quintile 5).

Presence of mental or physical health problems was a measure of the respondent’s self-rated overall health and description of illnesses diagnosed by a health care provider. Diagnosed physical illnesses were asthma, fibromyalgia, arthritis, back pain, high blood pressure, chronic bronchitis, emphysema or chronic obstructive pulmonary disease, diabetes, heart disease or cardiac problems, and cancer. Diagnosed mental health illnesses were mood disorder (depression, bipolar disorder, or dysthymia) and anxiety disorder (phobia, obsessive-compulsive disorder, or panic disorder).

Access to a usual source of health care was assessed by use of health services, such as visits to a doctor’s office, family medicine groups or units in hospitals, or community medical clinics. Physical activity was measured by using the short form International Physical Activity Questionnaire (IPAQ) (24). Health-related physical activity data referred to the last 7 days and were grouped into categories of low, moderate, and intense. Daily fruit and vegetable intake was dichotomized into fewer than 5 servings a day and 5 or more servings a day on the basis of Canada’s Food Guide (25). Obesity, a body mass index (kg/m^2^) of 30 or greater, was defined according to the Canadian Guidelines for Body Weight Classification (26). Diabetes, hypertension, cancer, and heart disease were diagnosed by a health care professional.

The study considered 3 explanatory variables: 1) disparities in material deprivation, 2) accessibility of health care services, and 3) personal lifestyle habits. Disparities in material deprivation combined annual household income and the material deprivation index. Combining these 2 variables provided an opportunity to study the simultaneous influence of the individual-level income variable on the ecological-level material deprivation variable and vice versa. The low income/deprived index group consisted of people with an annual income less than $40,000 (ie, the lower two-fifths of the Canadian income distribution) residing in a socioeconomically deprived neighborhood (ie, quintiles 4 and 5 of the material deprivation index). The high income/privileged index denoted individuals with an annual income of $40,000 or more (upper three-fifths of the income distribution) residing in a privileged neighborhood (quintiles 1, 2 and 3 of the index).

Measurement of accessibility to health care services combined 2 variables: having a usual source of care and having a family physician. Responses were classified as “good” if both were accessible; “limited” if either was accessible, and “no” if neither was accessible.

The measure of personal lifestyle habit was derived by combining the variables of smoking, physical activity, and daily fruit and vegetable intake. A healthy personal lifestyle indicated the respondent was currently a nonsmoker, engaged in intense physical activity in the past 7 days, and consumed 5 or more servings of fruits and vegetables per day. Respondents with a combination of any 2 of the above options were categorized as average, and those with none or only 1 of the 3 were categorized as having an unhealthy lifestyle.

### Statistical analysis

All analyses were performed by using SAS, version 9.1.3 (SAS Institute, Inc). First, we tabulated the univariate frequency distribution of population characteristics, the main explanatory variables (disparities in material deprivation, access to health care services, and personal lifestyle habits), and the dependent variables. Second, we used the Rao-Scott χ^2^ test to analyze bivariate associations between explanatory and outcome variables; significance was set at *P* < .05. For the multivariate logistic regression analyses, adjusted odds ratio (OR) estimates and 95% confidence intervals (CIs) were reported; significance occurred if the 95% CI of an OR estimate did not include unity. Six multivariable logistic regressions were run, 1 for each dependent variable. The 3 main independent variables (ie, disparities in material deprivation, access to health care services, and healthy lifestyle habits) were simultaneously entered in the models. In addition, all models included sex (if applicable), age, education, language spoken at home, immigration status, obesity, and presence of physical or mental illnesses.

## Results

Almost half of the population (48.0%) had unhealthy lifestyle habits, nearly two-fifths (39.0%) had limited or no access to health care services, and 58.1% reported some form of material deprivation (low income/deprived index, 19.3% low income/privileged index, 21.5%; or high income/deprived index, 17.3%) (Table 1). Of the preventive health care measures examined, mammography, blood glucose screening, and Papanicolaou (Pap) test were the most frequently performed tests (78.5%, 77.6%, and 73.4%, respectively), followed by blood pressure screening (67.3%). Seasonal influenza vaccination was 47.7%, and FOBT was the least performed test (21.0%).

**Table 1:** Socioeconomic and Health-Related Variables, Participants (N = 10,726) in Survey of Chronic Diseases and Their Determinants, Montreal, Canada, 2012

Variable	Measure
**Socioeconomic Variable, Unweighted %**	
**Sex**	
Male	44.8
Female	55.2
**Age, y**	
15–24	12.5
25–34	18.5
35–44	18.5
45–54	16.9
55–64	15.8
65–74	10.7
≥75	7.1
**Language spoken at home**	
French	55.6
English	20.6
Other	23.8
**Education**	
Less than secondary degree	16.0
Secondary degree to less than university	36.6
University degree or more	47.4
**Residency**	
Canadian (born in Canada)	64.5
Immigrant (resided in Canada ≥10 y)	21.3
Immigrant (resided in Canada <10 y)	14.3
**Annual household income, $**	
Less than 20,000	16.8
20,000–39,999	24.0
40,000–59,999	17.9
60,000–79,999	11.9
≥80,000	29.3
**Material deprivation index^a^**	
Most privileged (quintile 1)	21.0
Privileged (quintile 2)	21.2
Average (quintile 3)	20.3
Deprived (quintile 4)	20.0
Most deprived (quintile 5)	17.5
**Health Variable, Weighted % (95% CI)**	
**Mental health problem^b^**	
Yes (self-rated and/or diagnosed)	13.4 (12.7–14.1)
No (neither self-rated nor diagnosed)	86.6 (85.9–87.3)
**Presence of physical health problem^c^**	
Yes (self-rated and/or diagnosed)	47.8 (46.8–48.8)
No (neither self-rated nor diagnosed)	52.2 (51.2–53.2)
**Has access to usual source of health care^d^**	
Yes	84.6 (83.9–85.4)
No	15.4 (14.7–16.1)
**Has a family physician**	
Yes	64.8 (63.9–65.7)
No	35.2 (34.3–36.1
**Smoking status**	
Currently smoking	18.9 (18.2–19.7)
Currently not smoking	81.1 (80.3–81.8)
**Physical activity (past 7 days)**	
Low	23.1 (22.3–23.9)
Moderate	39.6 (38.6–40.5)
Intense	37.3 (36.4–38.3)
**Daily fruit and vegetable intake**	
Fewer than 5 servings	59.0 (58.1–60.0)
5 Servings or more	41.0 (40.0–41.9)
**Obesity (body mass index, kg/m^2^)**	
No (<30)	84.3 (83.6–85.1)
Yes (≥30)	15.7 (14.9–16.4)
**Diabetes**	
No	93.4 (93.0–93.9)
Yes	6.6 (6.1–7.1)
**Hypertension**	
No	83.0 (82.3–83.7)
Yes	17.0 (16.3–17.7)
**Cancer**	
No	98.0 (97.8–98.3)
Yes	2.0 (1.7–2.2)
**Heart disease**	
No	92.0 (91.5–92.6)
Yes	8.0 (7.4–8.5)
**Main Explanatory Variables, Weighted % (95%CI)**	
**Disparities in material deprivation (household income, material deprivation index^a^)**	
Low income / deprived (quintiles 4 and 5)	19.3 (18.6–20.1)
Low income / privileged (quintiles 1,2, and 3)	21.5 (20.7–22.4)
High income / deprived (quintiles 4 and 5)	17.3 (16.6–18.1)
High income / privileged (quintiles 1,2, and 3)	41.8 (40.8–42.7)
**Access to health care services (has usual source of care/family physician)**	
Good accessibility (both)	61.0 (60.1–61.9)
Limited accessibility (either)	28.0 (27.1–28.9)
No accessibility (neither)	11.0 (10.4–11.7)
**Healthy lifestyle habits (not smoking, intense physical activity, consuming ≥5 servings fruits or vegetables daily)**	
Unhealthy (none or 1)	48.0 (47.0–49.0)
Moderately healthy (any 2)	38.2 (37.2–39.2)
Healthy (all 3)	13.9 (13.2–14.6)
**Preventive Health Measures Variable (Weighted Percentage [95%CI])**	
Mammogram (last 2 years)^e^	78.5 (76.5–80.5)
Blood glucose test (last 3 years)^f^	77.6 (76.3–78.8)
Papanicolaou test (last 3 years)^g^	73.4 (72.0–74.7)
Blood pressure measurement (less than 1 year ago)^h^	67.3 (66.2–68.4)
Seasonal influenza vaccination (past 12 months)^i^	47.7 (46.0–49.3)
Fecal occult blood test (last 2 years)^j^	21.0 (19.6–22.4)

Abbreviation: CI, confidence interval.

a Material deprivation index is derived by principal component analysis based on the education, employment, and income characteristics of the smallest geographic area for which all census data were available. The index represents a continuous score. However, it is divided into quintiles to represent the most privileged (quintile 1 [best education, employment, and income]) to the most deprived (quintile 5 [least education, employment, and income]).

b Mood disorder (depression, bipolar disorder, mania, or dysthymia, including manic depression) and anxiety (phobia, obsessive-compulsive disorder, or panic disorder).

c Asthma, fibromyalgia, arthritis, back pain, high blood pressure, chronic bronchitis, emphysema or chronic obstructive pulmonary disease, diabetes, heart disease or cardiac problem, and cancer.

d A clinic or doctor’s office, family medicine groups or family medicine units in hospitals, or community clinics.

e Women aged 50 to 69 years with no cancer.

f Population aged 40 years or older with no diabetes and no heart diseases.

g Women aged 20 to 64 years with no cancer and no hysterectomy.

h Population aged 18 years or older with no hypertension and no heart diseases.

i Population aged 60 years or older as well as younger than 60 years with chronic diseases.

j Population aged 50 to 74 years with no cancer.

Use of preventive health services increased as material deprivation declined, access to health care improved, and lifestyle habits became healthier (Table 2). Results showed a significant bivariate association between all the study variables, except for FOBT (*P* = .10), blood glucose screening (p-value=0.70), and blood pressure screening (*P* = .06) and disparities in material deprivation, and between FOBT (*P* = .29) and vaccination (*P* = .09) and personal lifestyle habits. Specifically, the proportion of Pap tests was significantly higher among respondents in the high income/privileged index category (80.4%), those with good access to health care services (80.3%), and those with healthy lifestyle habits (79.6%) than among people in the remaining categories in each of these variables. Similar results were reported for mammography and blood pressure screening. Additionally, the proportion of FOBT (23.5%), blood glucose screening (86.9%), and influenza vaccination (51.7%) were substantially higher among populations with favorable access to health care services than among those with less access. Finally, the proportion of blood glucose screening (81.0%) was significantly elevated in respondents with healthy lifestyle habits.

**Table 2:** Association Between Receipt of Screening Tests and Seasonal Influenza Vaccination and the Main Explanatory Variables^a^, Survey of Chronic Diseases and Their Determinants, Montreal, Canada, 2012

Main Explanatory Variables^b^	Papanicolaou Test	Mammogram	FOBT	Blood Glucose Test	Blood Pressure Measurement	Influenza Vaccination
	Percentage (95% Confidence Interval)					
**Disparities in material deprivation (household income/material deprivation index)**						
Low income /deprived index (quintiles 4 and 5)	62.3 (58.8–65.8)	70.0 (64.7–75.3)	19.1 (15.9–22.3)	75.9 (72.9–78.9)	65.7 (63.1–68.3)	41.8 (38.2–45.5)
Low income /privileged index (quintiles 1,2, and 3)	67.4 (63.9–70.9)	77.0 (72.6–81.4)	21.7 (18.6–24.8)	77.5 (74.7–80.3)	67.2 (64.7–69.8)	51.2 (47.9–54.4)
High income /deprived index (quintiles 4 and 5)	74.6 (71.3–78.0)	80.0 (74.8–85.2)	17.1 (13.7–20.6)	76.5 (73.2–79.8)	64.5 (61.9–67.2)	39.4 (34.7–44.1)
High income /privileged index (quintiles 1, 2, and 3)	80.4 (78.4–82.3)	82.0 (79.1–84.9)	21.9 (19.8–24.1)	77.9 (76.0–79.8)	68.5 (66.9–70.2)	50.4 (47.7–53.2)
*P* value^c^	<.001	<.001	.10	.70	.06	<.001
**Access to health care services (usual source of care/family physician)**						
Good (access to both)	80.3 (78.7–81.9)	83.1 (81.0–85.1)	23.5 (21.8–25.2)	86.9 (85.7–88.1)	78.5 (77.2–79.8)	51.7 (49.8–53.6)
Limited (access to one or the other)	66.1 (63.3–68.9)	64.4 (58.5–70.2)	15.3 (12.3–18.4)	59.3 (56.1–62.5)	56.3 (54.1–58.4)	37.6 (33.5–41.6)
No (access to neither)	52.3 (47.0–57.7)	56.0 (45.2–66.8)	7.2 (3.7–10.6)	44.7 (39.5–49.9)	45.2 (41.8–48.5)	30.3 (24.0–36.6)
*P *Value^c^	<.001	<.001	<.001	<.001	<.001	.001
**Personal lifestyle habits (smoking/physical activity/fruit and vegetable intake )**						
Unhealthy (≤1)	71.3 (69.2–73.4)	74.2 (71.0–77.4)	20.6 (18.6–22.7)	75.4 (73.5–77.2)	65.1 (63.5–66.7)	45.8 (43.4–48.1)
Moderate (any 2)	73.5 (71.3–75.9)	81.5 (78.5–84.6)	20.3 (18.1–22.6)	79.0 (77.0–80.9)	69.1 (67.4–70.8)	49.2 (46.5–52.0)
Healthy (all 3)	79.6 (76.2–83.0)	82.2 (77.7–86.8)	23.7 (19.8–27.6)	81.0 (77.9–84.1)	69.9 (67.0–72.7)	50.3 (45.5–55.1)
*P* Value^c^	.001	.001	.29	.003	.001	.09

Abbreviations: FOBT, fecal occult blood test.

a Main explanatory variables are disparities in material deprivation (household income and socioeconomic conditions at the neighborhood level), access to health care services (having a usual source of health care and having a family physician), and personal lifestyle habits (smoking cigarette, physical activity, and daily fruit and vegetable intake).

b All values are expressed as percentage (95% confidence interval) unless otherwise indicated.

c *P* values are based on the Rao-Scott χ^2^ test for bivariate association between each explanatory variable and each screening test.

For Pap test, mammography, and vaccination models, all 3 main explanatory variables (ie, disparities in material deprivation, access to health care services, and personal lifestyle habits) were significant overall (Table 3). In the blood glucose and blood pressure screening models, however, material deprivation was not significant overall. Only accessibility to health care was significant in the FOBT model. In the Pap test model, the high income groups, irrespective of material deprivation index, were more likely to receive a Pap test than those in the low income/deprived index group (OR, 1.38; 95% CI, 1.04–1.84 and OR, 1.85, 95% CI, 1.43–2.39, respectively). Similarly, women with good or limited access to health care services had higher odds of having the Pap test (OR, 3.85, 95% CI, 2.86–5.17 and OR, 2.02, 95% CI, 1.50–2.74 respectively) than women with no access. Finally, women with healthy personal lifestyle habits were 48% more likely (OR, 1.48; 95% CI, 1.11–1.96) to have a Pap test than women with unhealthy lifestyle habits. Odds of compliance with mammography guidelines were significantly higher among women who were in the least materially deprived group (low income/privileged [OR, 1.54; 95% CI, 1.00–2.38] and high income/privileged [OR, 1.94; 95% CI, 1.27–2.97]), irrespective of household income level, had better access to care services (OR, 4.08; 95% CI, 2.34–7.11), and had moderate (OR, 1.54; 95% CI, 1.13–2.10) or healthy personal lifestyle habits (OR, 1.57; CI, 1.03–2.39). The direction and significance of the 3 explanatory variables were also similar for participants receiving the seasonal influenza vaccination. The same was true for blood glucose and blood pressure models except for the disparities in the material deprivation variable, which did not attain significance. Finally, only accessibility to health care services attained significance in the FOBT model.

**Table 3:** Adjusted Odds^a^ of Obtaining a Screening Testand Seasonal Influenza Vaccination, by Explanatory Variable^b^, Survey of Chronic Diseases and Their Determinants, Montreal, Canada, 2012^c^

Variable	Screening Test, Adjusted Odds Ratio (95% Confidence Interval)					
	Papanicolaou Test	Mammography	FOBT	Blood Glucose Test	Blood Pressure Measurement	Influenza Vaccination
**Material deprivation (household income, material deprivation index^d^)**						
Low income/deprived index	1 [Reference]	1 [Reference]	1 [Reference]	1 [Reference]	1 [Reference]	1 [Reference]
Low income/privileged index	1.13 (0.87–1.49)	1.54 (1.00–2.38)	1.02 (0.74–1.41)	0.90 (0.67–1.22)	0.87 (0.71–1.07)	1.31 (1.04–1.66)
High income/deprived index	1.38 (1.04–1.84)	1.56 (0.95–2.56)	0.85 (0.59–1.22)	1.13 (0.82–1.56)	0.94 (0.77–1.15)	0.96 (0.71–1.29)
High income/privileged index	1.85 (1.43–2.39)	1.94 (1.27–2.97)	1.03 (0.76–1.39)	1.03 (0.78–1.36)	0.99 (0.83–1.19)	1.42 (1.12–1.80)
**Access to health care services (usual source of care, family physician)**						
Good (access to both)	3.85 (2.86–5.17)	4.08 (2.34–7.11)	4.40 (2.40–8.04)	7.61 (5.79–9.99)	3.53 (2.94–4.23)	1.91 (1.33–2.74)
Limited (access to one or the other)	2.02 (1.50–2.74)	1.40 (0.75–2.58)	2.75 (1.43–5.26)	1.88 (1.41–2.50)	1.55 (1.29–1.86)	1.27 (0.85–1.91)
No (access to neither)	1 [Reference]	1 [Reference]	1 [Reference]	1 [Reference]	1 [Reference]	1 [Reference]
**Personal lifestyle habits (smoking, physical activity, food consumption)**						
Unhealthy (≤1)	1 [Reference]	1 [Reference]	1 [Reference]	1 [Reference]	1 [Reference]	1 [Reference]
Moderate (any 2)	1.10 (0.91–1.32)	1.54 (1.13–2.10)	1.00 (0.81–1.23)	1.17 (0.97–1.43)	1.24 (1.09–1.42)	1.16 (0.98–1.38)
Healthy (all 3)	1.48 (1.11–1.96)	1.57 (1.03–2.39)	1.13 (0.85–1.50)	1.47 (1.11–1.95)	1.27 (1.06–1.53)	1.34 (1.05–1.72)

Abbreviation: FOBT, fecal occult blood test.

a Controlled for sex (if applicable), age, education, language spoken at home, immigration status, obesity, and presence of physical and mental illnesses.

b Main explanatory variables are disparities in material deprivation, accessibility to health care services, and personal lifestyle habits.

c All values are adjusted odds ratios (95% confidence interval).

d Material deprivation index is derived by principal component analysis based on the education, employment, and income characteristics of the smallest geographic area for which all census data were available. The index represents a continuous score. However, it is divided into quintiles to represent the most privileged (quintile 1 [best education, employment, and income]) to the most deprived (quintile 5 [least education, employment, and income]).

## Discussion

We proposed and analyzed the independent and concurrent impact of disparities in material deprivation, access to health care services, and personal lifestyle habits on screening practices and disease prevention in a sample of 10,726 of the Montreal population in 2012. The relationship between the household income variable and the material deprivation index variable with engagement in clinical preventive services was documented (9,12,27). However, to our knowledge, the combined effect of these 2 variables on the likelihood of undergoing screening was not studied previously. Our study investigated this effect and provided an insight into relationships among variables. Namely, having a Pap test was significantly associated with high household income even if the woman had a deprived material index classification (ie, resided in a socioeconomically disadvantaged neighborhood), whereas mammography and influenza vaccination were significantly associated with a privileged material index classification (ie, resided in a privileged neighborhood) even among people with low household incomes. This finding may reflect differences in the delivery mechanism, ease of accessibility, and social expectation of receiving these preventive care measures. Quebec has well-organized public mammography and influenza vaccination programs. The Quebec Breast Cancer Screening Program invites women approaching age 50 to receive a free mammogram and follows up with them thereafter. Influenza vaccination begins every fall with mass media announcements, and several outreach campaign facilities are designated to serve at-risk populations. Public availability of these services enhances their accessibility, and continuous monitoring develops a sense of social expectation for prevention. There are, however, no corresponding Pap test campaigns for the target population, so cervical cancer screening is left to the discretion of women and their health care providers.

In general, the prevalence of having screening tests reported in this study did not vary substantially compared with other countries. For instance, the proportions of women undergoing mammography in the United States (72.5%) (7) and Europe (77.5%) (28) were comparable to the mammography rate in Montreal (78.5%). Also, the proportion of women having Pap test in Montreal (73.4%) was similar to the US rate (80.5%) (7) but lower than France’s rate (89.1%) (10). On the other hand, the proportion of people having FOBT screening for colorectal cancer (21.0%) was substantially lower in Montreal than in the United States (43.3%) (7) or Ontario (58.4%) (27). This difference may be partly attributable to the definition of colorectal cancer screening used in TOPO, which included FOBT only, whereas other studies also asked for endoscopy or colonoscopy (7,27). Finally, the proportion of blood glucose screening was higher in Montreal (77.6%) than in in the United States (31.0%) (14) but similar to Ontario (67.3%) (27).

TOPO is a population survey and may suffer from several limitations. For instance, because the data are based on self-reported information, they may be subject to recall bias. Additionally, the low response rate to TOPO could indicate selection bias if a particular characteristic of the sample is misrepresented. However, neither of these limitations presented a threat to the quality of our data, because distributions of several sociodemographic variables were comparable to those of the census data. Furthermore, there were no major differences between the distribution of the variables presented in Table 1 and those of Canadian Community Health Survey data (data not presented; available upon request). The cross-sectional nature of the study design does not let us infer any causal relationship between exposure and outcome variables. Finally, we considered personal lifestyle as an independent variable; however, evidence suggests that lifestyle choices may depend on broader socio-environmental factors (29). In this respect, the “two steps to prevention” approach advocates lifestyle as an intermediate variable between social determinants of health and access to prevention (30).

In summary, we found that disparities in material deprivation, access to health care services, and personal lifestyle habits had simultaneous and independent effects on the likelihood of receiving screening tests and influenza vaccination. Furthermore, the data provided insight on the differential effect of household income (an individual measure) and the material deprivation index (an ecological measure) on having a mammogram, Pap test, and seasonal influenza vaccination. This finding may indicate that high household income compensates for poor access to screening services when there are no community outreach prevention programs and may encourage public health practitioners to establish screening campaigns in underprivileged or socioeconomically disadvantaged neighborhoods to enhance public participation in disease prevention, thereby reducing disparities in health.
